# Policy review on health operational funds channelling in achieving maternal and child health programme in Eastern Java in Indonesia

**DOI:** 10.1186/1471-2458-14-S1-P5

**Published:** 2014-01-29

**Authors:** Niniek Lely Pratiwi

**Affiliations:** 1 Center for Humanities, Health Policy and Community Empowerment, National Institute of Health Research Development, Surabaya, East Java 60176, Indonesia

## Background

The number 2556/Menkes/Per/XII/2011 Permenkes policy guidelines on Health Operational Funds Channelling (BOK) has been revised several times during the process of implementation since first launched in 2010 by Ministry of Health in Indonesia. BOK disbursement to local governments as a form of government responsibility, for the development of public health, is aimed at improving health promotion and prevention efforts. It is carried out in order to accelerate health achievement of the Millennium Development Goal (MDG 4 and 5). The purpose of the study was to provide information on BOK policy evaluation in achieving maternal and child health programmes.

## Materials and methods

This study employed secondary data collection, from the years 2009 – 2011. District health profile was collected and primary focus group discussion (FGD) was carried out with invited technical officers implementing at health districts and health centres as well as some management staff of the local government BOK.

## Results

BOK policy launch from 2010 to 2011 showed that it was still slow at reducing maternal mortality and improving infant nutrition based on a review of data profiles in Sampang, Gresik and Sidoarjo districts. There were lacks of: 1) commitment of local government in developing strategies which prioritise maternal and child health programmes in the form of regional action plan innovation; 2) oversight, accountability controls, preventive and promotive activities of BOK especially in health centres located far from the county centre; 3) accountability on target, given the data attainment and child health neonatal visit coverage. It was found that BOK fund beneficiaries were more on health care providers, and not directly on the public.

## Conclusions

There is a need for commitment in the preparation of maternal and child health programmes with innovative movement, empowering local communities with the concept of social learning culture. Furthermore, the influence of the environment plays a very large role in shaping the character of the individual, past experience, as well as previous experience of parents can affect a community's social learning.

